# An Antisense Circular RNA Regulates Expression of RuBisCO Small Subunit Genes in *Arabidopsis*

**DOI:** 10.3389/fpls.2021.665014

**Published:** 2021-05-24

**Authors:** He Zhang, Shuai Liu, Xinyu Li, Lijuan Yao, Hongyang Wu, František Baluška, Yinglang Wan

**Affiliations:** ^1^Hainan Key Laboratory for Sustainable Utilization of Tropical Bioresources, College of Tropical Crops, Hainan University, Haikou, China; ^2^Environment and Plant Protection Institute, Chinese Academy of Tropical Agricultural Sciences, Haikou, China; ^3^State Key Laboratory of Tree Genetics and Breeding, Chinese Academy of Forestry, Beijing, China; ^4^College of Biological Sciences and Technology, Beijing Forestry University, Beijing, China; ^5^Institute of Molecular and Cellular Botany, Bonn University, Bonn, Germany

**Keywords:** antisense RNA, across-genic RNA, circular RNA, RBCS, *Arabidopsis thaliana*, expression regulation

## Abstract

Circular RNA (circRNA) is a novel class of endogenous long non-coding RNA (lncRNA) and participates in diverse physiological process in plants. From the dataset obtained by high-throughput RNA sequencing, we identified a circRNA encoded by the sense strand of the exon regions spanning two RuBisCO small subunit genes, *RBCS2B* and *RBCS3B*, in *Arabidopsis thaliana*. We further applied the single specific primer-polymerase chain reaction (PCR) and Sanger sequencing techniques to verify this circRNA and named it *ag-circRBCS* (*antisense and across genic-circular* RNA *RBCS*). Using quantitative real-time PCR (qRT-PCR), we found that *ag-circRBCS* shares a similar rhythmic expression pattern with other *RBCS* genes. The expression level of *ag-circRBCS* is 10–40 times lower than the expression levels of *RBCS* genes in the photosynthetic organs in *Arabidopsis*, whereas the *Arabidopsis* root lacked *ag-circRBCS* expression. Furthermore, we used the delaminated layered double hydroxide lactate nanosheets (LDH-lactate-NS) to deliver *in vitro* synthesized *ag-circRBCS* into *Arabidopsis* seedlings. Our results indicate that *ag-circRBCS* could significantly depress the expression of *RBCS*. Given that *ag-circRBCS* was expressed at low concentration *in vivo*, we suggest that *ag-circRBCS* may represent a fine-tuning mechanism to regulating the expression of *RBCS* genes and protein content in *Arabidopsis*.

## Introduction

Circular RNA (circRNA) is a class of covalently closed single-stranded RNA molecules that lack 5′ caps and 3′ poly(A) tails that generated by back-splicing events in eukaryotic cells (Wang et al., 2014). In 1976, Sanger et al. (1976) detected circRNAs in *Solanum lycopersicum* and *Gynura* as viroids by electron microcopy. With significant progress of high-throughput sequencing technologies and bioinformatic tools, scientists have verified that circRNAs are ubiquitous and abundant in eukaryotes, including in archaea (Danan et al., 2012), human (Jeck et al., 2013), *Arabidopsis* (Ye et al., 2015), and zebrafish (Shen et al., 2017). Several online databases have been constructed for deep analysis of circRNAs, including PlantcircBase (Chu et al., 2017), circBase (GlaŽar et al., 2014), CIRCpedia (Zhang et al., 2016), circRNADb (Chen et al., 2016), and AtCircDB (Ye et al., 2017). Chu et al. (2020) analyzed these published databases and identified feature differences between plant circRNAs and animal circRNAs. GT/AG or CT/AC dinucleotides are general canonical splicing signals in animals and plants, but the proportions of circRNAs with non-canonical splicing signals were shown to be variable across 12 different plant species. Plant circRNAs show great variety, and based on genomic features, they can be classified into 10 unique types, namely, e-circRNA, ei-circRNA, i-circRNA, ie-circRNA, u-circRNA, ue-circRNA, ui-circRNA, ig-circRNA, igg-circRNA, and ag-circRNA (e, i, u, g, ig, and ag represent exon, intron, UTR region, genic region, intergenic region, and across-genic region, respectively) (Chu et al., 2020).

CircRNAs may regulate gene expression using different mechanisms at transcriptional, post-transcriptional, and translation levels. For example, circSEP3 and circSMARCA5 may form an R-loop with a host DNA locus and regulate gene expression or inhibit the DNA damage repairing (Conn et al., 2017; Xu et al., 2021). In this case, the circRNA, CDR1, regulates target gene expression by sequestering miR-7, and circPan3 stabilizes IL-13 mRNA subunits, leading to the maintenance of stem cells (Zhong et al., 2019; Zhu et al., 2019). Endogenous circRNA containing an internal ribosome entry site (IRES) can be translated *in vivo* (Wang and Wang, 2015; Zhang et al., 2020), and circRNA–protein complexes have been reported (Schneider et al., 2016). It is still unclear whether the plant and animal circRNAs share similar mechanisms for regulation of gene expression. For example, CDR1 contains 63 conserved miR-7 binding sites and sequesters miRNA. However, there is still no evidence in plant cells to support such a sequestration-based transcription inhibition mechanism. To date, no plant circRNA with an IRES has been reported to have peptide translation.

Additionally, plant circRNAs play important functional roles in various biological processes and in different environmental stresses (Zhang et al., 2020). In *Arabidopsis*, 1,583 and 36 circRNAs were shown to be differentially expressed under heat and drought stress, respectively. In rice (*Oryza sativa*), 27 exonic circRNAs (6 up-regulated and 21 down-regulated) were found to be differentially expressed between normal and phosphate-starvation condition (Ye et al., 2015). Moreover, various environmental stresses, including dehydration, low nitrogen, drought, cold, heat, copper, salt, calcium, low phosphorus, and pathogen invasion, can cause alteration of circRNA expression in different species (Zhang et al., 2020), such as rice (Ye et al., 2015), tomato (*S. lycopersicum*) (Wang et al., 2018), wheat (*Triticum aestivum*) (Ren et al., 2018), soybean (*Glycine max*) (Lv et al., 2020), and maize (*Zea mays*) (Ghorbani et al., 2018). Lately, the amount of circRNA datasets has sharply increased, suggesting that plant circRNAs may have important roles in different biological processes. However, their function roles and regulation mechanisms require more experimental investigation and verification. For example, the overexpression of *circGORK* (guard cell outward-rectifying K^+^-channel) confirmed its role in drought tolerance (Zhang et al., 2019), and the overexpression of lariat-derived circRNAs suggested that circRNAs altered developmental phenotypes (Cheng et al., 2018).

RuBisCO is the most abundant enzyme on the Earth, and it is also solely responsible for all carbon fixation via photosynthetic assimilation of atmospheric CO_2_ (Bracher et al., 2017; Hayer-Hartl and Hartl, 2020). Therefore, it is the crucial enzyme for both feeding humanity and controlling the climate on Earth (Caetano-Anollés, 2017; Baluška and Mancuso, 2020; von Caemmerer, 2020). In higher plants and green algae, RuBisCO is composed of eight small subunits (RBCS) encoded by *RBCS* multigene family in the nuclear genome and eight large subunits (RbcL) encoded by a single *RbcL* gene in the chloroplast genome (Huang et al., 2020), denoted as RbcL_8_S_8_ (Bracher et al., 2017). For synthesis of the RuBisCO holoenzyme, both genes need to be expressed coordinately (Makino and Suzuki, 2012). RBCS subunits have been shown to influence the catalytic efficiency, CO_2_ specificity, assembly, activity, and stability of RuBisCO (Yamada et al., 2019), whereas the large subunits contain the active sites for catalytic activity. Therefore, the precise regulation of genes of the expression of large and small subunits is critical in the maintaining and regulation of RuBisCO activities.

In our previous study, we generated a circRNA database for etiolated and de-etiolation *Arabidopsis* seedlings (accession number: SUB3747127) (Liu et al., 2019). Among this dataset, a small group of circRNAs encoded by *RBCS* attracted our attention. Our RNA sequencing results suggested that these circRNAs were encoded by the sense strand of DNA, with complement sequence to the *RBCS3B* and *RBCS2B* mRNAs. Therefore, we verified these circRNA sequences and analyzed their spatial and temporal expression. We also used the delaminated layered double hydroxide lactate nanosheets (LDH-lactate-NS), a proven nanotransporter for intact plant cells (Bao et al., 2017; Song et al., 2019), to deliver the *in vitro* synthesized circRNA into *Arabidopsis* seedlings to investigate their biological functions.

## Results

### Identification of circRNA in *Arabidopsis* Seedlings

**Figure 1:**
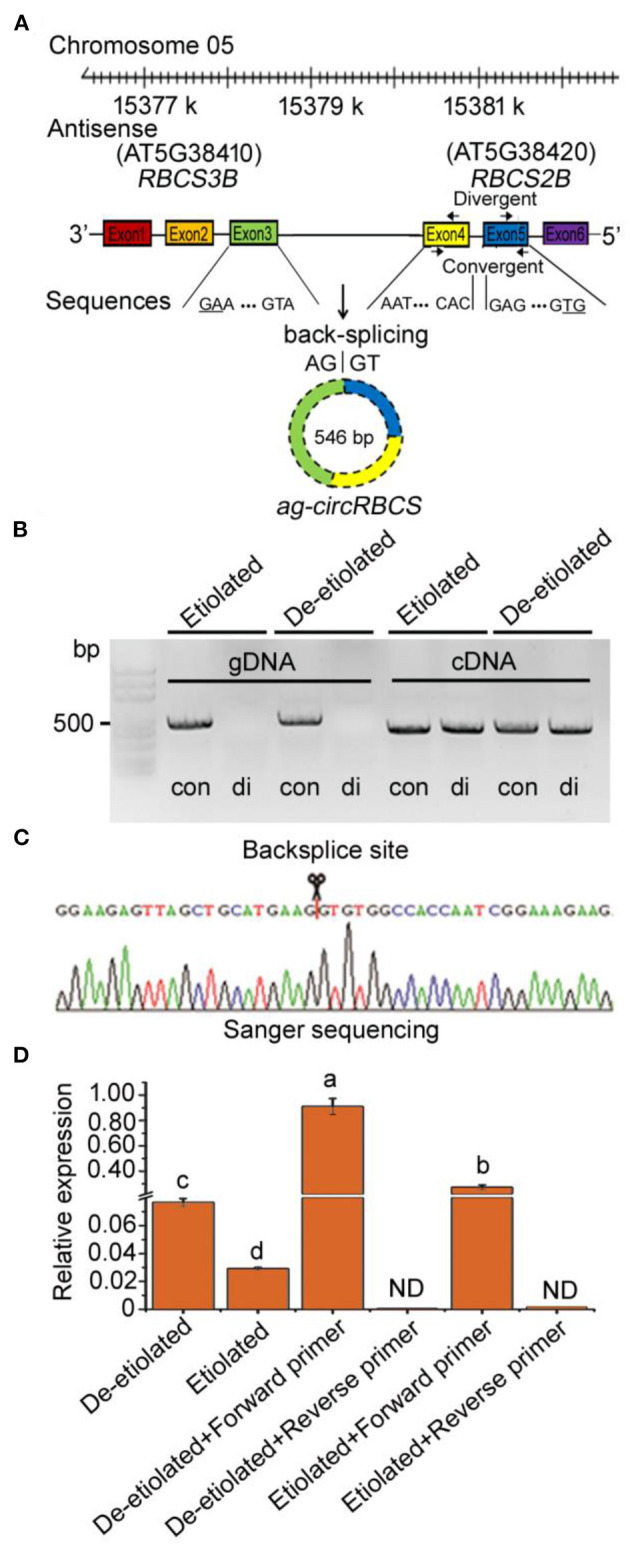
Identification of circular RNA from *Arabidopsis* seedlings. **(A)** *ag-circRBCS* is encoded by three exonic regions, exon3 of *RBCS3B* and exon4, 5 of *RBCS2B*. **(B)** Divergent primers successfully amplified circular RNAs in cDNA but failed in genomic DNA. Convergent primers worked on both cDNA and genomic DNA. con, convergent primers; di, divergent primers. **(C)** Sanger sequencing further confirmed head-to-tail back-splicing. **(D)** The circular RNA was confirmed as an antisense RNA *via* the single specific primer-PCR. All the data were analyzed for significant differences using ANOVA with Duncan's test. Different lowercase letters represent statistical significances of *p* < 0.05 (*n* = 3). ND, not detected.

From our previously reported strand-specific RNA sequencing database, we identified a small group of antisense circRNAs encoded by the sense strand of exon regions across RuBisCO small subunit genes in *Arabidopsis thaliana*, which we named *ath_circ_362-366* (Supplementary Figure 1). We designed pairs of convergent and divergent primers for polymerase chain reaction (PCR) to confirm the existence of these circRNAs. The divergent primers were designed across back-splice junctions, whereas the convergent primers were used as controls to validate linear sequences (Figure 1A). In the PCR reactions carried out with gDNA templates, only the convergent primers led to a positive band. Meanwhile, the divergent primers clearly indicated the existence of circRNAs using cDNA templates (Figure 1B). We tried to confirm the existence of *ath_circ_362-366* (data not shown), but only the primer pairs for *ath_circ_364* led to positive results (Supplementary Table 1).

Sanger sequencing based on these PCR products indicated that the *ath_circ_364* reflected a gene sequence encoded by three exonic regions, exon3 of *RBCS3B* and exon4, 5 of *RBCS2B*, meaning that this was a cross genic circRNA. Therefore, we named it *ag-circRBCS* (antisense and *across genic-circular* RNA *RBCS*) (Figure 1A). *ag-circRBCS* had a canonical site where the splice site was flanked by GT/AG and was formed by joining a splice donor to an upstream splice acceptor (Figure 1C).

### *ag-circRBCS* Is an Antisense RNA

Our RNA sequencing database suggested that *ag-circRBCS* had a higher expression in de-etiolated plants than in etiolated *Arabidopsis*, which was confirmed by quantitative real-time PCR (qRT-PCR) (Figure 1D). We further used the single specific primer-PCR (SSP-PCR) to define this strand-specific sequence. After multiple rounds of SSP-PCR, the cDNA contents of target sequences amplified with a complementary primer should be higher than before amplification. Therefore, a qRT-PCR assay followed SSP-PCR to indicate strand-specific cDNA sequences. In this study, we designed a forward primer that was complementary to the antisense sequence and the reversed primer as the control (Supplementary Table 2). After 35 rounds of SSP-PCR, the content of PCR products using the forward primer was much higher than those using the reverse primer, and in fact, the latter was almost undetectable. This result confirmed that *ag-circRBCS* reflected the antisense sequence of template DNAs. In addition, the de-etiolated seedlings had a much higher concentration of this than the etiolated seedlings (Figure 1D).

### Tissue-Specific and Rhythmic Expression of *ag-circRBCS*

**Figure 2:**
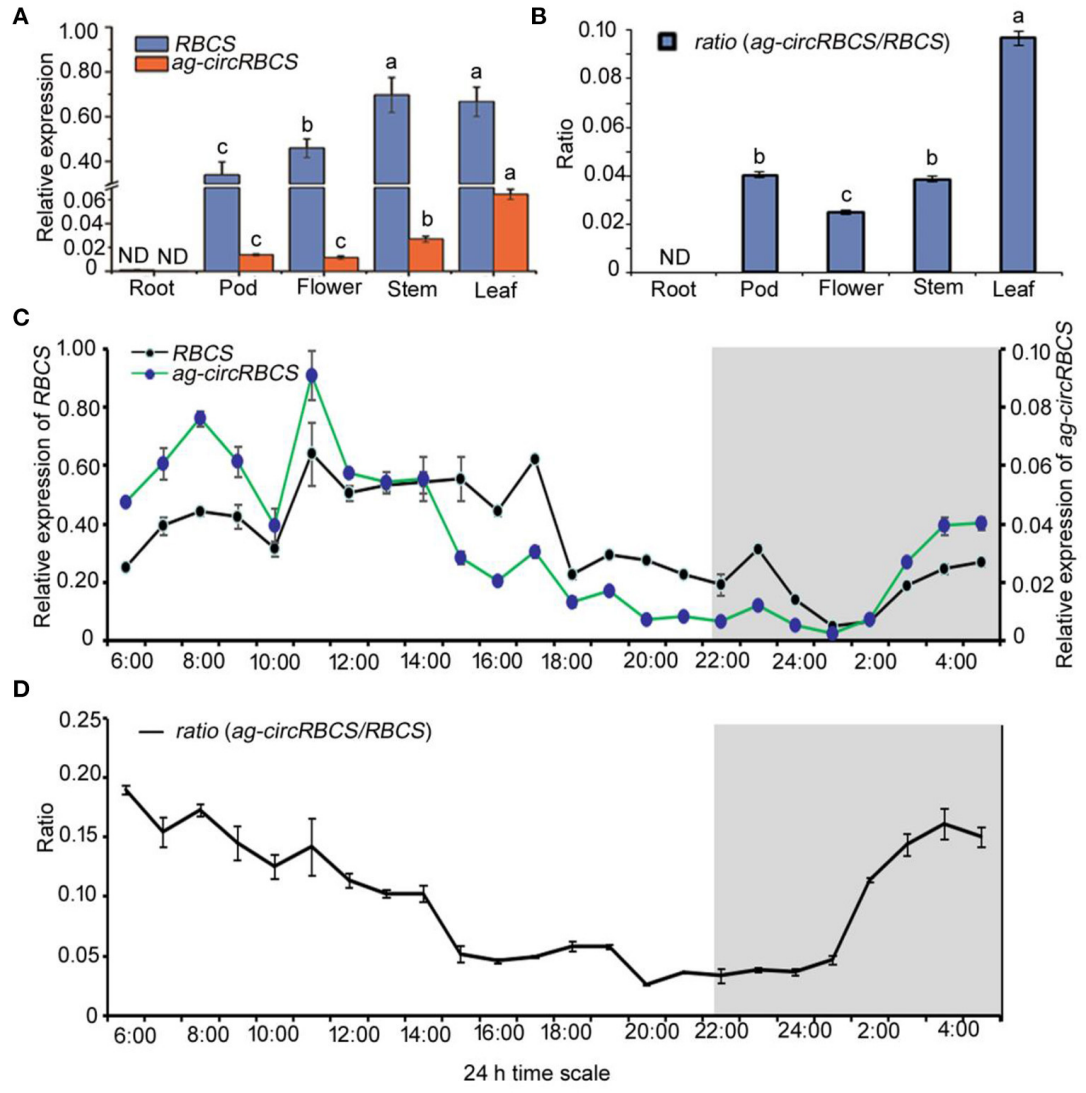
The expression profile of *RBCS* and *ag-circRBCS*. **(A)** The relative expression levels of *RBCS* and *ag-circRBCS* in different tissues. **(B)** The expression ratio of *ag-circRBCS* to *RBCS* in different tissues. *Actin* was used as an internal control. **(C)** The expression levels of *RBCS* and *ag-circRBCS* in different time points. **(D)** The expression ratio of *ag-circRBCS* to *RBCS* in different time points. *Actin* was used as an internal control. Data are expressed as mean ± standard deviation from three independent experiments. All the data were analyzed for significant differences using ANOVA with Duncan's test. Different lowercase letters represent statistical significances of *p* < 0.05 (*n* = 3) of RBCS, ag-circRBCS, and ratio, respectively. ND, not detected.

To investigate the relationship of the transcription level of *ag-circRBCS* and its parental gene, *RBCS2B* and *RBCS3B*, we used the qRT-PCR to quantify their spatial and temporal expression profile in *Arabidopsis*. Divergent primers were again used to quantify the expression of *ag-circRBCS*, and convergent primers were applied to understand the expression level of parental genes. Since sequences of *RBCS2B* and *RBCS3B* were very similar, the PCR products generated using convergent primers did not allow us the distinction between *RBCS2B* and *RBCS3B*. Therefore, we termed these genes *RBCS*. In underground tissue, the expression of both *RBCS* and *ag-circRBCS* was undetectable. In aerial tissues, *RBCS* in stems and leaves were not significantly differentially expressed, whereas the expression level of *RBCS* in pods and flowers was significantly lower than that in stems and leaves. Among all these tissues, the *ag-circRBCS* expressions level was the highest in photosynthetic tissue, namely, in the leaves. The expression of *ag-circRBCS* gradually decreased in stems, pods, and flowers. Moreover, the expression of *RBCS* was much higher than the expression of *ag-circRBCS*. In pods, flowers, stems, and leaves, the *RBCS* expression was 24.8, 40.2, 25.9, and 10.3 times higher than *ag-circRBCS*, respectively (Figure 2A). Meanwhile, the ratio of *ag-circRBCS* to *RBCS* was the largest in the leaves. This ratio was gradually decreased in pods, stems, and flowers (Figure 2B).

We further investigated the 24 h rhythmic expression pattern of *ag-circRBCS* in *Arabidopsis* seedlings. *Arabidopsis* seedlings were cultured under a routine long day illumination, e.g., 16 h in light and 8 h in dark, with illumination from 6:00 to 22:00. The profile of 24-h expression of both *RBCS* and *ag-circRBCS* showed similar typical periodic pattern (Figure 2C). The expression peak of *ag-circRBCS* occurred at 7:00 and 11:00, and the expression level of *ag-circRBCS* significantly decreased after 14:00. However, the expression level of *RBCS* remained relatively high between 11:00 and 17:00 and decreased after 18:00. Additionally, the peak of the ratio between the relative expression level of *ag-circRBCS* to *RBCS* occurred at 6:00, whereas the ratio of the off-peak occurred at 15:00–1:00. This ratio decreased slowly during the daytime and increased rapidly at night (Figure 2D).

### *In vitro* Artificial Synthesis, Identification of *ag-circRBCS*, and Tuned Parental Gene Expression and Function

**Figure 3:**
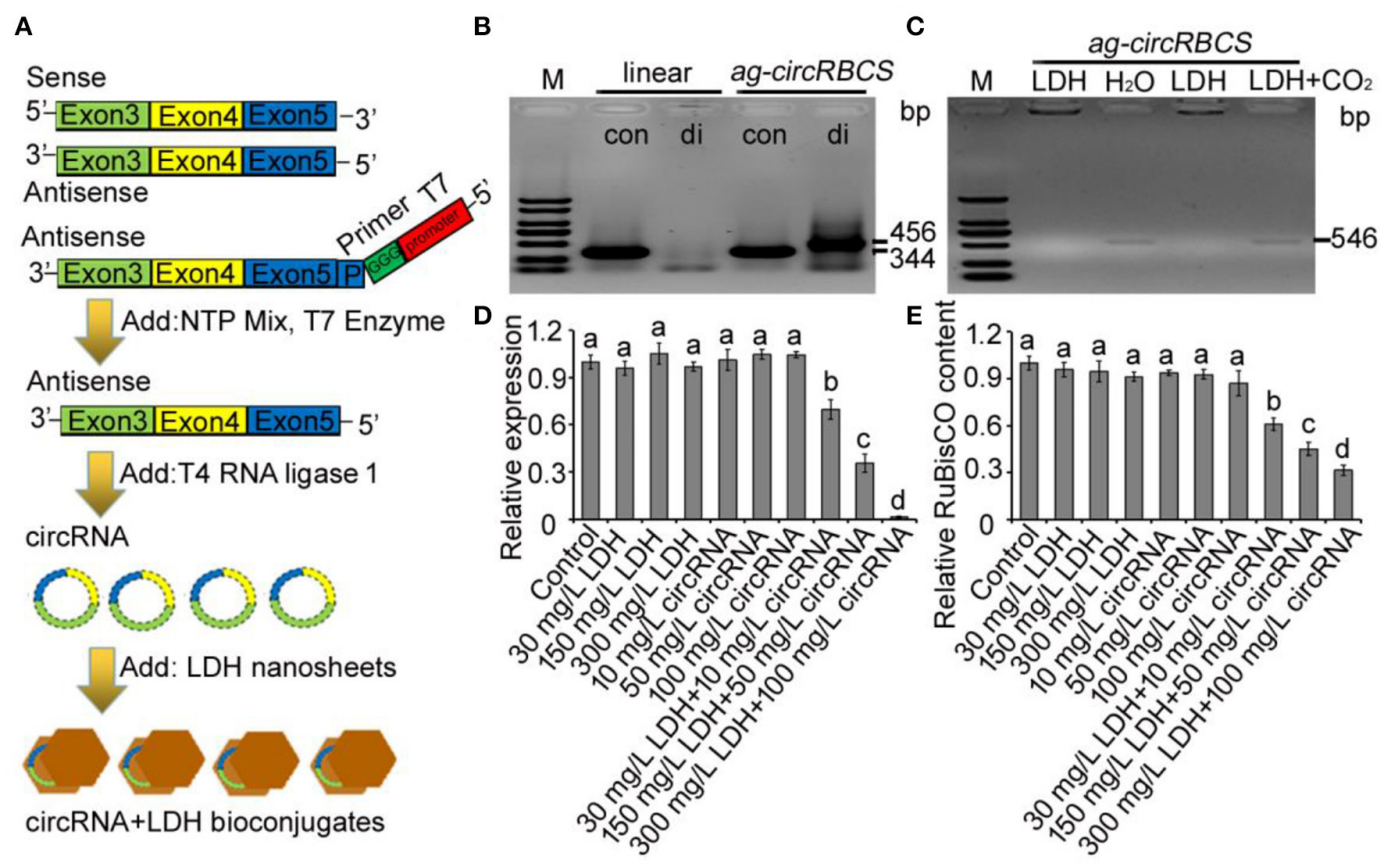
*ag-circRBCS* regulates *RBCS* gene expression. **(A)** Flowchart for artificial synthesis of *ag-circRBCS* and transported *via* layered double hydroxide lactate nanosheets (LDH-lactate-NS) *in vitro*. **(B)** The identification of synthesis *ag-circRBCS via* PCR. The convergent (con) and divergent (di) primers were used for analysis. **(C)** Analysis of the adsorption efficiency of LDH-lactate-NS *in vitro*. **(D)** Relative expression level of *RBCS* genes under different treatments. Numbers in **(B,C)** indicate the expected RNA length. **(E)** Analysis of the RBCS protein content *via* ELISA. M, marker; linear, synthesized single strand RNA before circulation; LDH, layered double hydroxide lactate nanosheets. *Actin* was used as an internal control. Data are expressed as mean ± standard deviation from three independent experiments. All the data were analyzed for significant differences using ANOVA with Duncan's test. Different lowercase letters represent statistical significance of *p* < 0.05 (*n* = 3).

*ag-circRBCS* was encoded by sense strand from the exonic regions of *RBCS2B* and *RBCS3B* genes. Obviously, the overexpression, knock-out, or knock-down of this circRNA would be expected to change its parental gene sequences, located on the antisense strand. Thus, we synthesized linear *ag-circRBCS* and circularized it *in vitro*. Next, LDH-lactate-NS was used to transport this synthesized circRNA into the leaf cells of *Arabidopsis* (Figure 3A). The linear antisense sequence of exon 3–5 of *RBCS3B* and *RBCS2B* was expressed in the T4 RNA ligase 1 system, and the successful synthesis and circulation of this RNA was validated by PCR with convergent and divergent primers (Figure 3B). By electrophoresis analysis, the PCR products with convergent primers could be detected in linear and cyclized RNA products, whereas the PCR products with divergent primers could only be found using cyclized RNA products, confirming the successful synthesis of *ag-circRBCS* (Figure 3B). The synthesized *ag-circRBCS* was then absorbed using LDH-lactate-NS *in vitro*. The efficiency of *ag-circRBCS* adsorption *via* LDH-lactate-NS was further investigated (Figure 3C). When the LDH was added to the synthesized *ag-circRBCS* at a weight ratio of 1:3, no *ag-circRBCS* repelled down. When natural air was bubbled into the LDH-ag-circRBCS solution for 1 h, the *ag-circRBCS* was replaced by CO_2_, and the obvious target band of *ag-circRBCS* appeared again (Figure 3C).

To investigate the effect of *ag-circRBCS* on the expression of parental or target genes, we used qRT-PCR to quantify the expression of *RBCS* genes and used enzyme-linked immunosorbent assay (ELISA) to quantify the RuBisCO protein contents with an RbcL antibody. When *Arabidopsis* seedlings were immersed in distilled water with LDH-lactate-NS or *ag-cricRBCS* separately, the expression level of *RBCS* was not affected, except in the solution with high LDH content. When the *Arabidopsis* seedlings were immersed in the solution with premixed LDH-lactate-NS and *ag-cricRBCS*, the expression of *RBCS* was significantly down-regulated (Figure 3D). Moreover, ELISA assay showed that the RuBisCO protein contents were down-regulated (Figure 3E).

## Discussion

In *Arabidopsis*, the *RbcS* gene family is further divided into two groups. Gene duplication and loss events of *RBCS* occurred during the evolution of *Arabidopsis* (Schwarte and Tiedemann, 2011). Therefore, three members of *RBCS* group B genes are homologous and tandemly distribute to chromosome 5. The expression of individual members of *RbcS* gene family has been shown to be separately regulated. For example, the *RBCS1A* is the only member distributed in root (Sawchuk et al., 2008), and the expression level of *RBCS1A, RBCS2B*, and *RBCS3B* is regulated by light signals, except *RBCS1b* (Dedonder et al., 1993). The tissue-specific expression and rhythmic expression of *ag-circRBCS* and its parental genes provide further clues for understanding the functional role of *ag-circRBCS*. *ag-circRBCS* was co-expressed with its parental genes, whereas both had their highest expression in photosynthetic tissues, coinciding with previous analysis of *RBCS* mRNA levels (Suzuki et al., 2009a,b). The rhythmic expression of *ag-circRBCS* and *RBCS* had similar profiles, too. The expression of *RBCS* had a circadian rhythm, such as maximally accumulated during subjective day and dropping to the lowest levels in the early evening (Choudhary et al., 2016). Our results agreed with this previous study and further suggested that the ratio of *ag-circRBCS*/*RBCS* expression reached a peak value in midnight (2:00). The rhythmic changes of the *ag-circRBCS*/*RBCS* expression ratio suggested that the *ag-circRBCS*/*RBCS* is not a simple by-product of *RBCS* expression.

**Figure 4:**
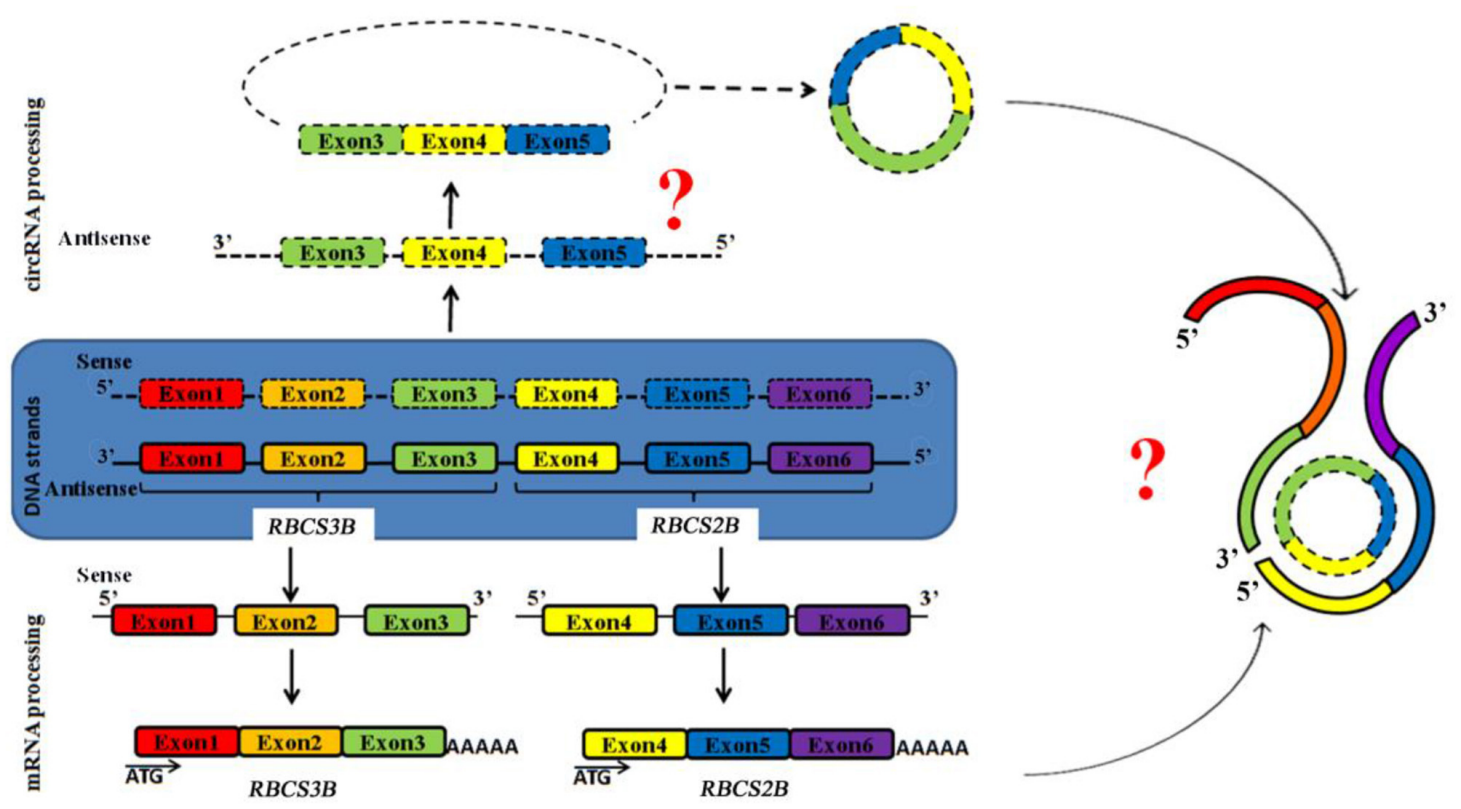
Schematic model to show the expression of both strands of genomic DNA of *RBCS3B* and *RBCS2B* genes. Square frames exhibit the exon regions. Two question marks indicate two critical steps: edition of antisense RNA and function of this antisense circRNA.

*ag-circRBCS* has a unique and novel structure according to known antisense RNAs. It is encoded by the exon region from the sense DNA strand of two neighboring genes. This RNA should be synthesized from an antisense pre-RNA with cross-genic sequences, and the splicing and back-splicing processes occur exactly as in sense pre-RNA (Figure 4). Thus, the expression of both antisense and sense strands was regulated by similar but unknown mechanisms. *ag-circRBCS* is not a by-product of *RBCS* expression, and its function needs to be further investigated.

Antisense RNA is an efficient means of regulating the expression of endogenous and foreign genes in eukaryotes (Xu et al., 2018). However, only a few functional antisense RNAs in plants have been reported. For example, a natural antisense RNA was shown to be involved in the regulation of MADS AFFECTING FLOWERING4 (MAF4) in *Arabidopsis* (Zhao et al., 2018). The overexpression of an antisense RNA of the maize gene ZmRLK7 in *Arabidopsis* resulted in regulating plant architecture and organ size formation (He et al., 2020). An antisense RNA may regulate target gene expression using different mechanisms. For example, antisense long non-coding RNAs (lncRNAs) may form L-loops with genomic DNA (Tan-Wong et al., 2019) or act as competing endogenous RNAs (ecRNA) (Tay et al., 2014). In this study, we did not investigate these mechanisms, but rather used a proven nanotransporter, LDH-lactate-NS, to deliver *in vitro* synthesized *ag-circRBCS* into plant cells. Our results indicate that a high concentration of *ag-circRBCS* can significantly depress the expression of its target genes, *RBCS2B* and *RBCS3B*, and even alter the expression of the RuBisCO protein itself. Given that *ag-circRBCS* has only 1/10–1/40 the relative expression level to *RBCS*, we suggest that it may provide fine-tuning to the regulation of its parental genes, the *RBCS2B* and *RBCS3B*. Moreover, an antisense circRNA provides an RNA formation with high stability during its life cycle, giving it resistance to RNase R. This conclusion coincides with an early study on rice, where antisense *RBCS* down-regulate the expression level of RuBisCO (Makino et al., 1997).

In conclusion, we report here that a novel circRNA, *ag-circRBCS*, may have roles in the precise regulation of RuBisCO expression. A possible model for its expression and formation is presented in Figure 4. We believe that this unique structure of an RNA sequence will provide new avenues for a better understanding of non-coding RNAs in plants.

## Materials and Methods

### Plant Material Preparation

The wild-type *A. thaliana* ecotype Col-0 (Columbia-0) was used for experiments. Single seed was chosen for further experiment within three generations. Seeds were placed on 1/2 MS medium (half-strength Murashige and Skoog-containing) with 0.4% Phytagels. Afterward, the plates were incubated in light chambers at 22°C with a 16-/8-h light cycle at 120 μmol photons m^−2^ s^−1^. The 10-day-old *Arabidopsis* seedlings were used for rhythmic transcriptional level assessments of *RBCS* and circRNAs. *Arabidopsis* seedling leaves were sampled every 1 h for 24 h. For tissue-specific expression, plants were cultivated in mixed soil (vermiculite/nutrition soil = 2:1, v/v). The *Arabidopsis* seedlings were germinated and grown in a 16-h light/8-h dark photoperiod at 22°C. The 30-day-old *Arabidopsis* seedlings were used to assessing the specific transcriptional level of *RBCS* and circRNAs.

### RNA Extraction and cDNA Synthesis

Total RNA was extracted, and DNA contamination was removed by using the E.Z.N.A. Total RNA Kit (OMEGA, GA, USA). DNase I was added to the total RNA extraction for 10 min at 37°C to digest DNA and then kept at 65°C for 10 min to inactivate the DNase I. The quality and concentration of total RNA samples were assessed by 1.5% agarose gel electrophoresis and NanoDrop 2000 spectrophotometer. The purified RNA served as a template for synthesizing first-strand cDNA using the TransScript One-Step gDNA Removal kit and cDNA Synthesis Super Mix (TransGen, Beijing, China).

### Quantitative Real-Time PCR

qRT-PCR was performed using the SYBR® Premix Ex Taq™ II (Perfect Real Time) (TaKaRa, Dalian, China) in a typical 20 μl PCR mixture including 10 μl of SYBR® Premix Ex Taq™ II, 1 μl of template cDNA, and 0.2 μM of each PCR primer, with double distilled water up to 20 μl. Samples were mixed gently and centrifuged briefly to collect droplets. The cycling conditions were 95°C for 5 min, followed by 40 cycles at 95°C for 20 s, 53°C for 20 s, and 72°C for 30 s, and samples were run on a Real-Time PCR Detection System CFX96 (Bio-Rad, CA, USA). All of the relative gene expression levels were calculated using the 2^−ΔΔCt^ method, with *A. thaliana actin* (*AT3G18780*) used as an internal control. The primers used for qRT-PCR are listed in Supplementary Table 2.

### Single Specific Primer-PCR

For SSP-PCR, cDNA of de-etiolated or etiolated *Arabidopsis* seedlings was used as template, and divergent forward primer and divergent reverse primer were added for first round of PCR, respectively. These fragments were amplified by first round of PCR using the Pyrobest DNA polymerase (TaKaRa, Dalian, China) for 35 cycles in a 50 μl reaction with these primers (Supplementary Table 2). The reactions were amplified using the qRT-PCR mix using the “Quantitative real-time PCR” program on a Biorad thermocycler.

### Quantification of RuBisCO Content

For RuBisCO quantification, *Arabidopsis* seedlings were suspended in phosphate buffered solution (PBS, pH 7.4, 0.15 M) on ice. Then, the RuBisCO in the supernatant was quantified using a plant RuBisCO ELISA kit (Jiangsu Meimian Industrial Co., Ltd., Jiangsu, China) according to the manufacturer's instructions. Briefly, the supernatant was added to a 96-well plate and combined with horseradish peroxidase (HRP)-labeled RbcL enzyme antibody at 37°C for 1 h. After washing 5 times, 3,3′,5,5-tetramethylbenzidine (TMB) was quickly added to the reaction, and then sulfuric acid solution was quickly added to terminate the reaction. The absorbance of reaction mixtures was then measured at 450 nm with the ELx800 microplate reader (Bio Tek, EV, UK). The concentration in each well was then calculated based on a standard curve. The amounts of RBCS were calculated from the RuBisCO holoenzyme and the ratio of molecular mass between RBCS and RbcL (Makino and Suzuki, 2012; Suganami et al., 2020).

### *In vitro* Synthesis and Circularization of circRNA

Large quantities of antisense linear circRNA were synthesized in Dongxuan jiyin (Jiangsu dongxuan jiyin Jiangsu Technology Co., Ltd., Jiangsu, China) by T7 RNA polymerase using the TranscriptAid T7 High Yield Transcription Kit (Thermo Scientific, NY, USA). The sequences are listed in Supplementary Table 1. After DNase I treatment for 10 min at 37°C, RNA was purified by LiCl precipitation and then treated with T4 polynucleotide kinase (New England Biolabs, MA, USA) in the presence of ATP. Finally, the linear RNA was circularized with T4 RNA ligase 1 (New England Biolabs, MA, USA) for 16 h at 16°C. Any residual linear RNA was eliminated by RNase R for 10 min at 37°C. After electrophoresis, RNA in bonds with a size of about 500 bp was collected by E.Z.N.A. ploy gel RNA extraction kit (OMEGA, GA, USA). Finally, the collected circRNA was precipitated and concentrated *via* equal volume ethanol.

### Validation of circRNAs

Divergent and convergent PCR primers were designed for circRNA validation (Supplementary Table 2). First-strand cDNA was synthesized using divergent primers or convergent primers. The PrimeSTAR® Max DNA Polymerase (Takara, Dalian, China) was used for cDNA amplification with PCR to detect circRNA templates. The PCR mixture included 25 μl PrimeSTAR Max Premix (2×), 1 μl of template cDNA, and 1 μl of each PCR primer, with double distilled water up to 50 μl. The cycling conditions used were 95°C for 5 min, followed by 40 cycles at 95°C for 20 s, 53°C for 20 s, and 72°C for 30 s on a Biometra T1 Thermocycler. Then, Sanger sequencing was performed on all PCR products to validate their sequence. Before performing Sanger sequencing, the PCR products were electrophoresed on 1% agarose gel and collected by GeneJET gel extraction kit (Thermo Scientific, Lithuania).

### Adsorption and Desorption of LDH

Mixtures of circRNAs and LDH with weight ratios of 1:3 were incubated at 25°C for 1 h in airtight tubes. After that, the mixtures were transferred to DNase-, RNase-, and DNA-free 6-well tissue culture plates (Thomas Scientific, NJ, USA) and exposed in air at 25°C for 1 h. The adsorption effect was detected via 1% agarose gel electrophoresis with Tris/Borate/EDTA buffer at 5 V/cm.

### Functional Analysis of circRNA in *Arabidopsis* Seedlings

For function analysis, 10-day-old seedlings were infiltrated at 6-well tissue culture plates with 3 ml of different circRNA:LDH solutions, namely, 300 mg/L LDH + 100 mg/L circRNA, 150 mg/L LDH + 50 mg/L circRNA, and 30 mg/L LDH + 10 mg/L circRNA. Then, 100 mg/L circRNA, 50 mg/L circRNA, 10 mg/L circRNA, 300 mg/L LDH, 150 mg/L LDH, and 30 mg/L LDH were used as negative controls, respectively. Afterward, seedlings were transferred to 22°C conditions with a 16-h light/8-h dark photoperiod for 3 days. Then, the seedlings' leaves were stored at −80°C for future use.

### Statistical Analyses

Mean and standard deviation (SD) are displayed as representative values for data in the figures. Analysis of variance (ANOVA) with Duncan's test was used to assess statistical significance, which was done with SPSS v10, unless otherwise noted. *p* < 0.05 was regarded as statistically significant.

## Data Availability Statement

The datasets presented in this study can be found in online repositories. The names of the repository/repositories and accession number(s) can be found in the article/Supplementary Material.

## Author Contributions

HZ and SL did most of the experimental works and wrote the manuscript. SL did the database analysis. XL and HW synthesized the LDH. LY did the 24 h analysis. FB helped in the manuscript writing and supported this project. YW supervised this project. All authors contributed to the article and approved the submitted version.

## Conflict of Interest

The authors declare that the research was conducted in the absence of any commercial or financial relationships that could be construed as a potential conflict of interest.

## Supplementary Material

Supplementary Figure 1 Library preparation for circRNA sequencing.

Supplementary Table 1 Sequences of circular RNAs encoding the antisense *RBCS* genes.

Supplementary Table 2 Primers used in this study.
