# Supplementary figures and images for: An Antisense Circular RNA Regulates Expression of RuBisCO Small Subunit Genes in Arabidopsis
Source: Front Plant Sci. 2021 May 24;12:665014. doi: 10.3389/fpls.2021.665014 (PMC8181130; doi:10.3389/fpls.2021.665014)

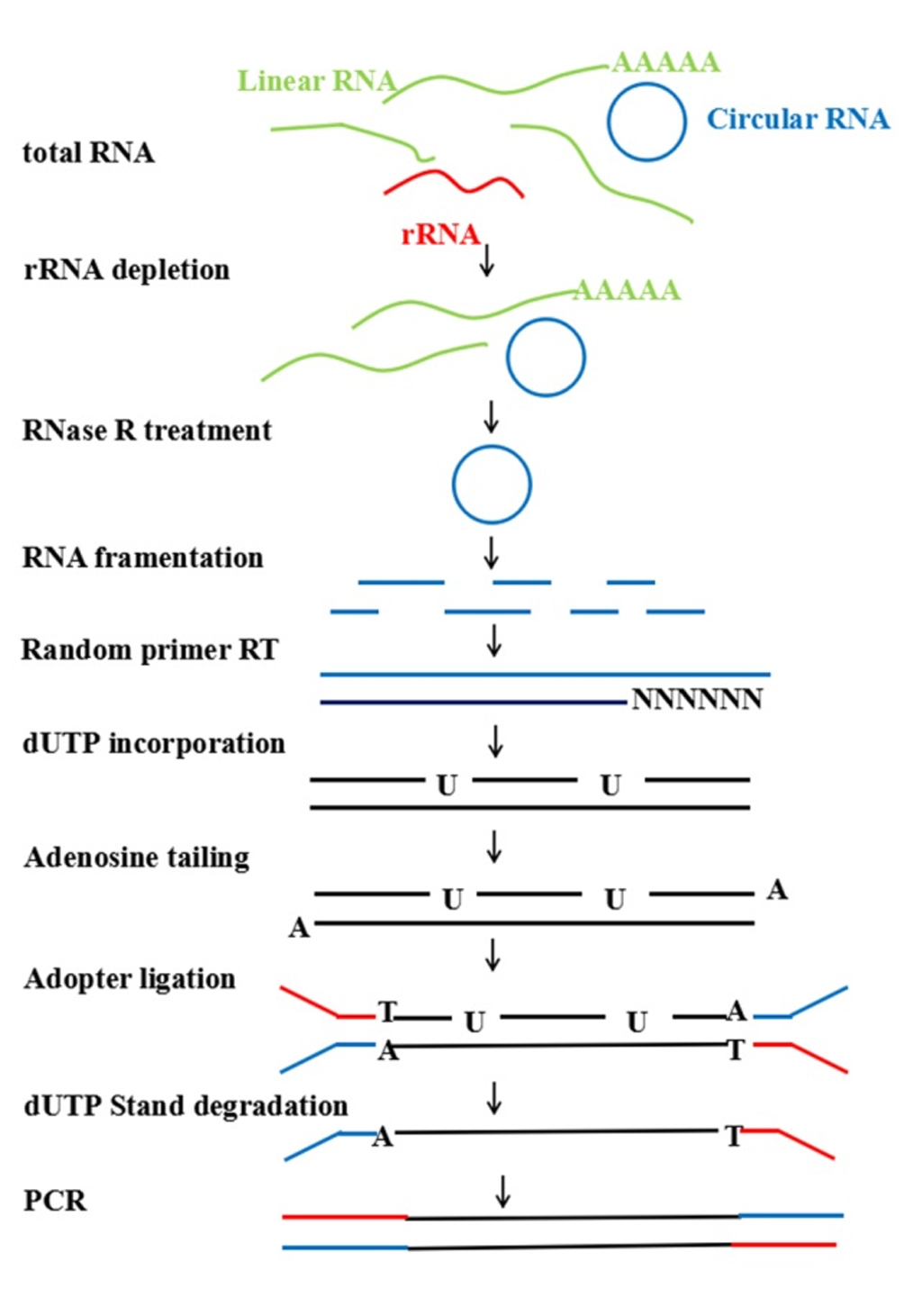

Supplement: Supplementary Figure 1 — Library preparation for circRNA sequencing. [file Image_1.JPEG]
